# Genome-wide map of human and mouse transcription factor binding sites aggregated from ChIP-Seq data

**DOI:** 10.1186/s13104-018-3856-x

**Published:** 2018-10-23

**Authors:** Ilya E. Vorontsov, Alla D. Fedorova, Ivan S. Yevshin, Ruslan N. Sharipov, Fedor A. Kolpakov, Vsevolod J. Makeev, Ivan V. Kulakovskiy

**Affiliations:** 1 Vavilov Institute of General Genetics, Russian Academy of Sciences, GSP-1, Gubkina 3, Moscow, Russia 119991; 2 BIOSOFT.RU Ltd, Russkaya 41/1, Novosibirsk, Russia 630058; 3 Institute of Computational Technologies, Siberian Branch of the Russian Academy of Sciences, Akad. Rzhanova 6, Novosibirsk, Russia 630090; 4 Novosibirsk State University, Pirogova 2, Novosibirsk, Russia 630090; 5 Engelhardt Institute of Molecular Biology, Russian Academy of Sciences, GSP-1, Vavilova 32, Moscow, Russia 119991; 6 Moscow Institute of Physics and Technology (State University), 9 Institutskiy per, Dolgoprudny, Russia 141700; 7 Institute of Mathematical Problems of Biology RAS-the Branch of Keldysh Institute of Applied Mathematics of Russian Academy of Sciences, Vitkevicha 1, Pushchino, Russia 142290

**Keywords:** Transcription factor binding sites, ChIP-Seq, Cistrome, Regulatory regions, Target genes, Human and mouse

## Abstract

**Objectives:**

Mammalian genomics studies, especially those focusing on transcriptional regulation, require information on genomic locations of regulatory regions, particularly, transcription factor (TF) binding sites. There are plenty of published ChIP-Seq data on in vivo binding of transcription factors in different cell types and conditions. However, handling of thousands of separate data sets is often impractical and it is desirable to have a single global map of genomic regions potentially bound by a particular TF in any of studied cell types and conditions.

**Data description:**

Here we report human and mouse cistromes, the maps of genomic regions that are routinely identified as TF binding sites, organized by TF. We provide cistromes for 349 mouse and 599 human TFs. Given a TF, its cistrome regions are supported by evidence from several ChIP-Seq experiments or several computational tools, and, as an optional filter, contain occurrences of sequence motifs recognized by the TF. Using the cistrome, we provide an annotation of TF binding sites in the vicinity of human and mouse transcription start sites. This information is useful for selecting potential gene targets of transcription factors and detecting co-regulated genes in differential gene expression data.

## Objective

Locations of genomic regions responsible for transcriptional regulation are valuable for many genomics and genetics studies, from analysis of gene regulatory networks to prediction of the functional impact of non-coding genetic variants. There are thousands of experimental data sets related to in vivo binding of human and mouse transcription factors (TFs), with chromatin immunoprecipitation followed by deep sequencing (ChIP-Seq) as the gold standard method. Many existing databases such as GTRD [1] or ReMAP [2] focus on systematic reprocessing and user-friendly access to published ChIP-Seq data.

By design, ChIP-Seq data provide information on cell-type specific binding. Binding profiles of the same TF can be quite different in different cell types or experimental conditions, and, for a particular transcription factor, it is not always feasible to separately analyze hundreds of individual data sets. Instead, a list of reproducible TF binding regions routinely identified as TF binding sites could be valuable for preliminary selection of putative target genes or for the discovery of key regulators for differentially expressed genes. For example, meta-clusters provided in GTRD are the genome segments bound by the studied TF across several data sets. For practical applications it is useful to have such constituent binding regions being separated into different reproducibility categories, and annotated with occurrences of transcription factor binding motifs, to highlight likely genuine binding sites of each particular TF. In particular, transcription factor binding sites in the vicinity of the transcription start site are of special interest allowing to identify putative TF target genes. Finally, it would be convenient to have available genomic coordinates of a TF binding map for each of several commonly used genome releases.

## Data description

Here we present the human and mouse cistromes [3], the genome-wide maps of regions bound by TFs, obtained through systematic analysis of ChIP-Seq data. The cistromes include data for 349 mouse and 599 human TFs. Cistromes provide an important information layer for detecting putative target genes of the corresponding TFs, for detecting regulators bound to known promoters and enhancers, and for intersection and enrichment analysis of various genomic features including regulatory sequence variants.

For each TF, the cistrome consists of sets of non-overlapping regions with assigned reliability categories. For convenience, we provide genome-wide (Table 1, Data set 1–4) and gene-centric (Table 1, Data set 5–8) maps for two major human (hg19, hg38) and mouse (mm9, mm10) genome assemblies [4, 5]. The genome-wide map contains global genomic coordinates of TF binding regions. The gene-centric map contains the relative locations of the nearest cistrome segments for each transcription start site (TSS).

**Table 1:** Overview of data files/data sets

Label	Name of data file/data set	File types (file extension)	Data repository and identifier (DOI or accession number)
Data set 1	cistrome_hg19.zip	Archive file (.zip) containing genomic regions files (.bed)	Figshare (10.6084/m9.figshare.7087697)
Data set 2	cistrome_hg38.zip	Archive file (.zip) containing genomic regions files (.bed)	Figshare (10.6084/m9.figshare.7087697)
Data set 3	cistrome_mm9.zip	Archive file (.zip) containing genomic regions files (.bed)	Figshare (10.6084/m9.figshare.7087697)
Data set 4	cistrome_mm10.zip	Archive file (.zip) containing genomic regions files (.bed)	Figshare (10.6084/m9.figshare.7087697)
Data set 5	cistrome2genes_hg19.zip	Archive file (.zip) containing tab-separated files (.tsv)	Figshare (10.6084/m9.figshare.7087697)
Data set 6	cistrome2genes_hg38.zip	Archive file (.zip) containing tab-separated files (.tsv)	Figshare (10.6084/m9.figshare.7087697)
Data set 7	cistrome2genes_mm9.zip	Archive file (.zip) containing tab-separated files (.tsv)	Figshare (10.6084/m9.figshare.7087697)
Data set 8	cistrome2genes_mm10.zip	Archive file (.zip) containing tab-separated files (.tsv)	Figshare (10.6084/m9.figshare.7087697)
Data file 1	cistrome_overview.xlsx	MS Excel file (.xslx)	Figshare (10.6084/m9.figshare.7087697)

### Cistrome aggregation and motif annotation

The initial set of TF binding regions from ChIP-Seq (the ChIP-Seq peaks) was extracted from GTRD (release 17 April 2017). GTRD provided ChIP-Seq peak calls from four different peak calling software (see Data file 1 for details) executed with default parameters. Using the approach described in [6], some data sets were excluded as unreliable. Then we applied BEDTools 2.26.0 [7] to merge the overlapping intervals from different experiments and ChIP-Seq peak callers. The resulting regions were classified into four reliability categories in the following manner:

A (the highest reliability, experimental and technical reproducibility): this group contains cistrome regions consisting of overlapping peaks detected in at least two experimental data sets and by at least two peak calling tools, i.e. supported by at least two experiments and at least two peak callers;

B (high reliability, experimental reproducibility): regions supported by at least two experiments;

C (medium reliability, technical reproducibility): regions supported by at least two peak callers.

For segments of A, B, and C sub-cistromes, it is required that each segment overlaps at least one ChIP-Seq peak from a data set that was accompanied by the experimental control data. All other reproducible segments fall into D category (limited reliability). The technical details of the cistrome construction and overall statistics of the cistrome are provided in the Data file 1 (see Table 1).

All the cistrome categories were annotated by predictions of TF binding sites with HOCOMOCO v11 [6] sequence motifs to obtain a subset of regions with genuine binding sites recognized by a particular TF.

Data for human hg38 and mouse mm10 genome assemblies was produced directly from GTRD peak calls, data for human hg19 and mouse mm9 assemblies was produced with liftOver (v353) [8].

A (the best constitutively bound sites) and joint ABC (the compromise) cistromes are the most informative. We used those along with the motif annotation to construct a gene-centric map of TF binding (Table 1, Data set 5–8) using the GENCODE [9] annotation (GTF, main annotation files) and PyRanges 0.0.13 [10]. For each TSS, the gene-centric map contains the absolute distance from a TSS to the nearest cistrome segment corresponding to binding of a particular TF.

## Limitations

- The cistrome lacks metadata regarding cell types, antibodies or experimental conditions. For studies of particular genes or particular binding sites, the user is advised to address a detailed database, such as GTRD.
- The cistrome coverage and reliability heavily depends on a volume of experimental data available for a particular TF. In the presented map, many TFs have very sparse maps with only a few bound regions or only low-reliability cistrome categories, or with no cistrome regions at all.
- For many TFs it was not possible to perform the motif annotation due to absence of reliable information on binding sequence preferences, the corresponding entries are explicitly marked in the gene-centric map.

## Abbreviations

TF: transcription factor
ChIP-Seq: chromatin immunoprecipitation followed by deep sequencing
TSS: transcription start site
